# Bacteria in the Atmosphere

**Published:** 1904-10-01

**Authors:** 


### The Hospital.

A Journal of

The Medical Sciences and Hospital Administration.

Vol. XXXVII.—No. 940. OCTOBER 1, 1904.

### Bacteria in the Atmosphere.

Some recent experiments by Dr. Mervyn Gordon, on the contamination of the air of rooms by bacteria proceeding from the respiratory passages of the persons occupying them, and especially of those speaking, singing, coughing, sneezing, or otherwise strongly projecting the expired air, which are fully described in the recently issued report of the Medical Officer of the Local Government Board, appear to throw a large amount of light upon what may be described as the mechanism of personal infection by vicinity, without direct contact. All estimations of the fouling of the air of inhabited rooms which had previously been made have rested upon chemical composition alone, and mostly upon the proportion of carbon-dioxide; but it is manifest that the air of such rooms must become fouled, not only by gaseous exhalations from the bodies of the occupants, but also by emanations which are particulate, and that, in the acts of coughing and sneezing more especially, liquid matter from the air passages must be sprayed into the atmosphere. It had been proved that persons suffering from phthisis might in this way diffuse the tubercle; bacillus which, indeed, had actually been recovered from the air of rooms that they had occupied; but no general means had been perfected of detecting in the air, say of public buildings, the quantity and quality of the particulate matter of possibly deleterious nature which had been shed into it by the persons assembled there, in a way analogous to that by which contaminating matter, of human or animal origin, is now readily discoverable in our water supplies.

Dr. Greenwood, the Medical Officer of Health for Blackburn, who anticipated Dr. Gordon in the publication of his results, although probably much subsequent to him as respects the date at which his inquiries were commenced, announced some weeks ago, in his report to the local authorities of the town, that he had exposed plates of gelatin and other nutrients in the schoolrooms before and during or after the time at which they were occupied by classes, and that the number of colonies developing upon such plates was enormously greater under the latter conditions; but he did not describe any investigation of the nature of the colonies, or of the relative abundance of different kinds of bacteria. Dr. Gordon, on the other hand, commenced his work by searching for some specific organism which was normal to human saliva, and sufficiently characteristic to be accepted as an evidence of its presence, and he found this in a streptococcus which he describes as streptococcus brevis, and which is commonly present in saliva to the extent of at least 10,000,000 in a cubic centimetre of the secretion. This organism is readily identified by the character, among others, that it produces a definite change of colour on culture under anaërobic conditions at 37° Centigrade in neutral red broth; and Dr. Gordon determined, by another set of experiments, that minute quantities of human saliva produce the same change by reason of the streptococci of this species which they contain.

In applying the knowledge thus gained for the purposes of experiment, Dr. Gordon, first in a small and subsequently in a large room, observed the effects of loud speaking in the distribution of droplets of the saliva of the speaker through space. Having ascertained, by means of Petri dishes charged with nutrient broth and distributed about the rooms employed, that the air contained in them did not hold either streptococcus brevis or any other streptococcus in suspension, he, by similar distribution of culture media during and immediately subsequent to oration there by a series of separate speakers, was able to demonstrate as a result of these orations a general dissemination of droplets of saliva throughout the air of the rooms, a distribution extending to a distance of 40 feet in front of the speakers, and of 12 feet behind them, these being the greatest at which the culture media were exposed. Flügge and others in Germany had previously made somewhat similar experiments, and had obtained similar results, from speakers whose mouths were first artificially contaminated with bacillus prodigiosus, and they, like Dr. Gordon, failed to discover evidence of contamination from merely quiet speaking, although after loud speaking, coughing, or sneezing, it was abundant. Dr. Gordon describes some observations, the results of which were little more than negative, with outside air collected in London and in Blackheath, and he infers from them, so far as they go, that salivary streptococci are comparatively seldom met with in the open air. A streptococcus which is of frequent occurrence, both in the open air and in the air of rooms, and which is also to be found in dust, can be easily distinguished from the streptococci characteristic of recent samples of saliva.

We are indebted to Mr. Shirley Murphy for the knowledge of the definite relation which has been found to exist between the holidays and the re-assembling of the London elementary schools and the diminution or increase of infectious diseases, and especially of diphtheria and scarlet fever. The investigations of Dr. Gordon appear to throw much light upon the probable mechanism, so to speak, of the increase, and to point to the speech of slightly affected children as a means by which pathogenetic bacteria may easily be conveyed to their classmates and playfellows. The diffusion of the salivary droplets appears to depend partly upon the original projecting force by which they are expelled, and partly upon the currents of air in the room itself; and Dr. Gordon makes the practical suggestion that downward ventilation might be of practical value in quickly removing from a room any salivary contamination which was liberated in it. It is evident that his inquiries have opened a way to investigations which will require to be extensively pursued. A small proportion of our old friend carbon dioxide will no longer be sufficient to justify the giving of a clean bill of health to a school-room.

